# Mixing Ability of Intercropped Wheat Varieties: Stability Across Environments and Tester Legume Species

**DOI:** 10.3389/fpls.2022.877791

**Published:** 2022-06-09

**Authors:** N. Moutier, A. Baranger, S. Fall, E. Hanocq, P. Marget, M. Floriot, A. Gauffreteau

**Affiliations:** ^1^INRAE, Institut Agro Agrocampus Ouest, Université de Rennes 1, UMR 1349 IGEPP, Le Rheu, France; ^2^INRAE, UE 0972 GCIE, Estrées-Mons, Péronne, France; ^3^INRAE, UE 115 Epoisses, Bretenière, France; ^4^Agri-Obtentions, Orsonville, France; ^5^INRAE, AgroParisTech, Université Paris Saclay, UMR 211 Agronomie, Thiverval-Grignon, France

**Keywords:** cereal, pea, faba bean, breeding, G×G×E interactions, land equivalent ratio (LER), producer/associate concept, mixtures

## Abstract

Cereal-legume intercrops are developed mainly in low input or organic farming systems because of the overyielding and numerous ecosystem services they provide. For this management, little advice is available for varietal choice and there are almost no specific breeding programs. Our study aimed to evaluate the mixing ability of a panel of bread wheat genotypes in intercropping and to assess the impact of environment and legume tester choice on this ability. We used partial land equivalent ratios (LERs) to assess the mixing ability of a genotype defined as the combination of its ability to maintain its own yield in intercropping (producer effect, LERw) and to let the mixed species produce (associate effect, LERl). Eight wheat genotypes and 5 legume testers (3 pea and 2 faba bean varieties) were grown in sole crop and in all possible binary intercrops in nine contrasting environments. A mixed model was used to evaluate the effects of wheat genotypes, legume testers, environments, and all the interactions among these 3 factors on LERw and LERl. The chosen wheat genotypes presented contrasting mixing ability, either in terms of producer effect (LERw) or associate effect (LERl). A strong negative correlation was observed between these two components of genotype mixing ability, with an increase in producer effect being generally associated with similar decrease in associate effect, except for three genotypes. The impact of environment on the producer and associate effects was limited and similar between genotypes. Legume tester had a significant effect on both LERw and LERl, making the choice of tester a major issue to reveal the producer or associate effects of wheat genotype. Although the 5 testers showed no significant differences in wheat genotype order for both producer or associate effects, they showed different competitiveness and ability to discriminate genotypes: faba bean was very competitive, resulting in low LERt and low capacity to discriminate wheat genotypes for their mixing ability. On the contrary, pea was less competitive, resulting in higher LERt and better capacity to discriminate wheat genotypes. In particular, the Hr varieties (Geronimo and Spencer) discriminated best the wheat genotypes. Consequences on the implementation of breeding programs for wheat varieties adapted to intercropping are discussed.

## Introduction

During the last 60 years, agriculture in industrialised countries has become more intensive, focusing on reduced numbers of main crops in shorter rotations on increasingly large sole crop plots. Higher yields were obtained thanks to the intensive use of mineral fertilisers and synthetic pesticides, which strongly impacted the environment (environmental degradation, resources depletion), agrosystem biodiversity, and human health (Tilman et al., 2002). The acknowledged need to move towards more sustainable and responsible agriculture and design more resilient arable cropping systems was advocated by Altieri et al. (2017) through agroecological principles, among which are (1) the diversification of the agroecosystem by increasing the biodiversity at landscape, farm, and field levels, over time and space, and (2) the optimised use of beneficial biological interactions that are naturally available in the agrosystem to maximise ecological services.

Intercropping, which corresponds to simultaneous cultivation of two or more crop species in the same field (Willey, 1979), strongly contributes to these principles. It is an old and widespread practice in many areas of the world (Anil et al., 1998). It has been largely abandoned in Europe following intensification but arouses a renewed interest in the context of transition from intensive to low-input systems (Malézieux et al., 2009).

Intercropping can provide higher, more secure, and stable yields than sole crops (Raseduzzaman and Jensen, 2017; Stomph et al., 2020), with less or no external inputs. It also improves soil conservation and fertility, and grain protein concentration of a cereal when intercropped with a legume (Gooding et al., 2007). It allows for better control of pests and weeds (Banik et al., 2006; Boudreau, 2013; Lopes et al., 2015) and reduces lodging (Chen et al., 2020). Intercropping derives these advantages from the ecological principles of complementarity, cooperation, competition, and compensation between crops, the so-called “4C approach” (Bedoussac et al., 2015; Justes et al., 2021). Intercropping usually brings together two (or more) species affected by different pests, showing contrasted root and/or aerial systems, displaying different sensitivities to low or high temperature and complementary requirements for natural resources (light, water, and/or nutrients) in time and/or space. An obvious example is cereal-legume intercrops: in which the use of soil mineral nitrogen by non-leguminous crops is complemented by atmospheric nitrogen fixation by leguminous crops.

In spite of these many potential advantages, the adoption of intercrops stays at low levels in Europe (apart in conservation agriculture and organic farming) due to remaining technical, economic and policy barriers to wider dissemination, as recently shown in the case of bread wheat and field pea intercrops (Mamine and Farès, 2020).

Just on the field scale, many factors may indeed influence intercrop services and performances: environmental conditions (rainfall, temperatures, soil fertility, etc.), crop management practices, such as species choice (Wendling et al., 2017), sowing densities and dates (Neumann et al., 2007; Pötzsch et al., 2019), spatial designs (Ndzana et al., 2014), fertilisation strategies (Yu et al., 2016), and availability of machinery settings (sowing, harvesting, and sorting).

Although the varietal choice within each species is likely to affect canopy traits, resource access, provided ecosystemic services and performance of the mixtures, there are only few publications on the varietal effect on intercropping. They often focused on specific performance or service of one of the species, and/or integrated a limited number of varieties from intercropped species in a limited number of environments: response to nitrogen application (Hauggaard-Nielsen and Jensen, 2001), nitrogen use efficiency (Tsialtas et al., 2018), disease control (Kinane and Lyngkjaer, 2002), quality and yield performance (Barker and Dennett, 2013; Baxevanos et al., 2017; Kammoun et al., 2021), biomass production (Streit et al., 2019; Li et al., 2020). Recently, reports considering larger varietal diversity are emerged (Haug et al., 2021).

Since very little data are available to date to assess the mixing ability of a given variety in intercrop, either for its capacity to produce (producer effect) or its ability to make the associated species produce (associate effect), farmers base their varietal choice on traits and performances evaluated in sole crop. This practice may be risky, since some of these traits and performances are not always predictive of those observed in mixtures (Hauggaard-Nielsen and Jensen, 2001; Moutier et al., 2018, 2021; Annicchiarico et al., 2019).

To consider adapted breeding methodologies targeting this cultivation practice (Annicchiarico et al., 2019; Sampoux et al., 2020), breeders need to be able to assess the mixing ability of genotypes belonging to a focal species both across environments and companion species/varieties. These companion species/varieties are below called testers for their potential to reveal the mixing ability of a genotype, by analogy to testers used to identify superior germplasm in hybrid-oriented breeding programs (Hallauer et al., 2010; Fasahat et al., 2016). Since an intercropped tester may also influence the mixing ability of a genotype because of its competitive and discriminatory power, breeders also need to find adequate testers to reduce the number of combinations studied.

The purpose of our study was to (i) identify the potential impact of wheat varietal choice on the productive and associated performances when wheat is mixed with different tester varieties from two grain legumes species (pea and faba bean), (ii) evaluate if the varietal mixing ability is stable across environments and testers, (iii) compare the capacity of contrasted tester legume species to discriminate stably wheat genotypes for their suitability for intercropping.

## Materials and Methods

### Plant Material and Experimental Design

Eight bread winter wheat (*Triticum aestivum*) genotypes, all early maturing (to ensure joint harvest with legumes), resistant to lodging, and partially resistant to main diseases (especially to yellow rust), were chosen according to yield potential (high vs. low), earliness in heading stage (early vs. mid early-mid late), and canopy height at heading stage (short vs. tall) in sole crop (SC). The genotypes covered all 8 possible combinations of the 3 previous traits (Table 1A).

**TABLE 1:** Phenological, architectural, and agronomic traits in sole crop (SC) of **(A)** the 8 winter bread wheat genotypes and **(B)** the 5 field pea and faba bean varieties involved in binary mixtures.

A. Wheat genotype							
		Yield potential		Earliness at heading stage		Height at heading stage	
				
		Expected^1^	*Observed* ^2^	Expected^1^	*Observed* ^2^	Expected^1^	*Observed* ^2^
		*q/ha*				*cm*
Flamenko		high	*50*	early	*May 14*	short	*80*
Geny			*49*		*May 14*	tall	*83*
Attlass			*48*	mid early–mid late	*May 18*	short	*75*
RE13003			*49*		*May 21*	tall	*87*

Forcali/Rebelde^3^		low	*37*	early	*May 12*	short	*74*
CF14336			*44*		*May 16*	tall	*88*
Renan			*41*	mid early–mid late	*May 19*	short	*80*
Ehogold			*41*		*May 19*	tall	*102*

**B. Legume varieties**							
	**Type^1^/Species**	**Flowering starting date (SC)**		**Plant height at harvest (SC)**		**Soil coverage power (SC)**	
				
		**Expected^1^**	***Observed* ^2^**	**Expected^1^**	***Observed* ^2^**	**Expected^2^**	***Observed* ^3^**
					***cm***		***%***

Fresnel	hr field pea	early	*April 22*	high	*81*	high	*37 (early stages) – 99 (late stages)*

Geronimo	Hr field pea	late	*May 19*	low	*75*	low (early stages) to high (late stages)	*20 (early stages) – 83 (late stages)*
		
Spencer	late	*May 20*	low	*75*	low (early stages) to high (late stages)	*19 (early stages) – 85 (late stages)*

Irena	Faba bean	early	*April 15*	low	*102*	low	*30 (early stages) – 70 (late stages)*
		
Olan		late	*April 21*	high	*120*	high	*33 (early stages) – 79 (late stages)*

*(A) ^1^Expected trait from pre- or post-certification trial data and/or from breeder/expert communication. ^2^Mean observed trait under SC conditions across 9 environments (this issue, organic or low inputs systems). ^3^The cultivar Forcali was replaced by Rebelde, showing the same varietal trait combination, in 2018 and 2019.*

*(B) ^1^Hr field pea varieties are highly responsive to photoperiod for their floral initiation, whereas hr field pea varieties are not.*

*^2^Expected trait from pre- or post-certification trial data and/or from breeder/expert communication.*

*^3^Mean observed trait under SC conditions across 9 environments (this issue).*

Five legume testers, i.e., 2 faba bean (*Vicia faba*) and 3 afila field pea (*Pisum sativum*) varieties, including 2 “Hr” varieties needing a minimal photoperiod to initiate flowering and 1 “hr” variety whose flowering initiation does not depend on photoperiod, were chosen according to their phenological (flowering starting date) and architectural (plant height at harvest and soil coverage power) traits to create different competition conditions with wheat in time and space (Table 1B). All the legume testers were late maturing in sole crop to ensure joint harvest with wheat.

All possible binary mixtures of the eight wheat genotypes with the five legume testers were considered, leading to 40 intercrop (IC) and 13 sole crop modalities.

Each trial contained two parts: one with the wheat SC, the pea SC, and the wheat-pea IC, i.e., a total of 35 treatments; the other with the faba bean SC and IC, i.e., a total of 18 treatments. This spatial distribution is aimed at suppressing the neighbouring effects of faba bean (in SC and IC) on the other species plots. In these two parts, the treatments (35 vs. 18) were distributed into 8–10 m^2^ microplots according to a complete randomised block design with three blocks and one replicate per block.

### Environments and Management Practices

**FIGURE 1:**
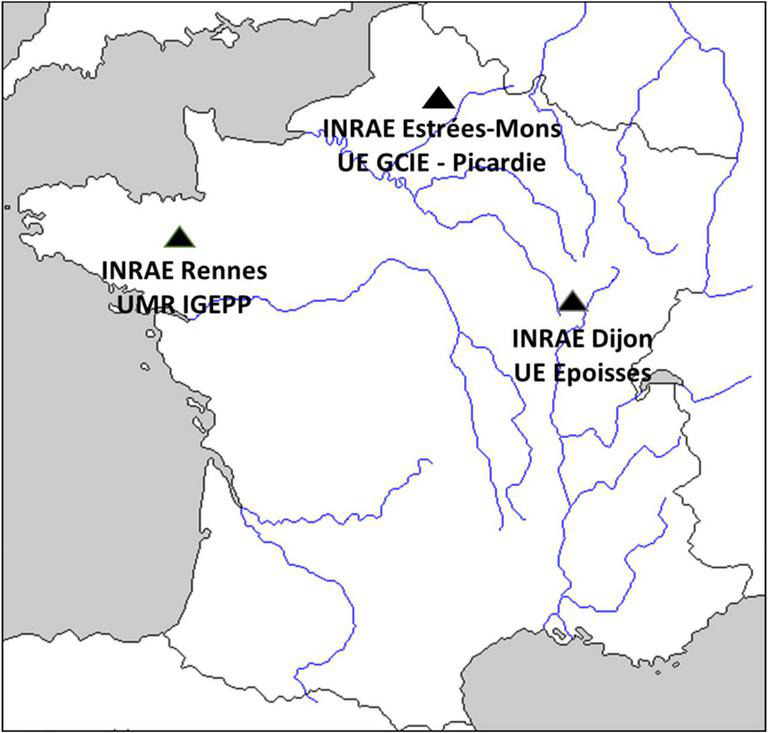
Location of the French National Research Institute for Agriculture, Food and Environment (INRAE) experimental sites of Dijon, Estrées-Mons and Rennes.

Nine trials (3 locations × 3 years) were conducted by INRAE (French National Research Institute for Agriculture, Food and Environment) from 2016/17 to 2018/19 seasons, in organic farming (Rennes, RE) or very low input systems (Estrées-Mons, EM and Dijon, DI; Figure 1).

The 3 locations were known to be contrasting in their soil characteristics (type and depth) and climatic conditions over the 10 years preceding the implementation of the trials (cumulative rainfall and distribution of precipitation over the growing season: minimum, medium, and maximum temperatures; Table 2).

**TABLE 2:** Agronomic and environmental characteristics (means in the 2006–2016 period) of the 3 French National Research Institute for Agriculture, Food and Environment (INRAE) experimental sites (Dijon, Estrées-Mons, and Rennes).

		Temperature (°C)					
Location	Climatic zone	Mean	Min	Max	Cumulated rainfall (mm)	Soil type	Soil depth
		
		On the growing season (October 21–July 20)					
INRAE Dijon (DI)	semi-continental	9.3 (8.2 to 10.6)	–10.1 (–20.2 to –4.7)	34.1 (31.8 to 37.7)	560 (470 to 660)	clay-loam to clay	moderately deep
INRAE Estrées-Mons (EM)	oceanic	9.0 (7.8 to 10.3)	–8.6 (–15.8 to –2.5)	31.8 (27.9 to 34.6)	490 (370 to 620)	loamy	moderately deep
INRAE Rennes (RE)	oceanic	10.3 (9.5 to 11.1)	–5.9 (–8.6 to –3.3)	32.1 (27.8 to 35.8)	590 (440 to 770)	loamy, beating	deep

The microplots were sown in the fall (from 21 October in DI and EM to 9 November in RE in autumn 2016) with a 6- to 8-row grain seed drill, spacing 13.5–20 cm between the rows, just after ploughing, and with grains fully mixed in the row for IC.

The wheat genotypes were sown at 300, 150, and 210 seeds/m^2^ in SC, IC with pea, and IC with faba bean, respectively. The hr pea genotype was sown at 80 and 60 seeds/m^2^ in SC and IC, respectively, while the Hr pea genotypes were sown at 40 seeds/m^2^ both in SC and IC, and the faba bean genotypes were sown at 28 and 21 seeds/m^2^ in SC and IC, respectively. The relative higher pea and faba bean sowing densities (75–100% of SC density) than that of wheat (50–70% of SC density) in the ratio was justified by higher competitiveness of wheat expected in the ICs, together with the aim of an expected balance of species in the harvest (Lithourgidis et al., 2011).

There were no or few chemical controls, and no fertiliser was spread on the crops (except for 30 units of nitrogen supplied at the end of March 2018 in EM). Weeds were mainly managed by mechanical weeding when needed. The harvest occurred in mid-July. The harvested grains of the two species were separated mechanically.

### Variables Under Study

Each year, for each sorted sample from the 159 microplots and for each of the species, the weight and the moisture were measured, and the gross yield was calculated.

The performance of each mixture component (either wheat or legume) in each trial and block was evaluated from the observed wheat or legume yields in SC and IC by partial land equivalent ratio (Crookston and Hill, 1979) as follows:


(1) $$\mathrm{LERw}=Y_{w\,\left(\mathrm{IC}\right)}/Y_{w\,\left(\mathrm{SC}\right)}\,\mathrm{and}\,\mathrm{LERl}=Y_{l\,\left(\mathrm{IC}\right)}/Y_{l\,\left(\mathrm{SC}\right)},$$

where LERw and LERl are the partial land equivalent ratios for wheat and legume, Y_w(IC)_ and Y_w(SC)_ are the yields of wheat in IC and SC, Y_l(IC)_ and Y_l(SC)_ are the yields of legume in IC and SC.

The performance of each mixture was then evaluated by its total land equivalent ratio (LERt) by the sum of the partial wheat and legume LER values as follows:


(2) LERt=LERw+LERl,

Total land equivalent ratio measures the total land area under SC (in ha) required to produce the same amount of grain as the wheat-legume IC in 1 ha.

This index allows comparing performances in IC relative to SC. If LERt > 1, environmental resources are used more efficiently by IC than by SC.

In our study, the mixing ability of a wheat genotype was defined both by its capacity to maintain its SC potential yield, i.e., to limit the loss of yield in IC compared to SC, and its ability to make the associated species produce. In each trial and block, the ability of a wheat genotype to maintain its yield when intercropped with legumes (producer effect) was estimated by the ratio of its yield in IC to its yield in SC, corresponding to LERw. Similarly, the ability of a wheat genotype to maintain the yield of the associated legume genotype (associate effect) was estimated by the ratio of legume yield when intercropped with this particular wheat genotype to the legume yield in SC, corresponding to LERl.

### Statistic Model

In order to identify the terms to be included in the analysis, the following model was first considered:


(3) Y=ijtkμ+G+iT+jE+tGT+ijGE+itTE+jtEBtk+GTE+ijtε,ijtk

where the LERw or LERl of the wheat genotype i intercropped with the legume tester j in the block k of the environment t, and the block k (Y_ijtk_) is decomposed in an overall mean (μ) and the effects of the wheat genotype i (G_i_), legume tester j (T_j_), environment t (E_t_) and all possible interactions between these three factors. The experimental design was also considered through the block effect in each environment (EB_tk_). A preliminary analysis of variance showed that all the terms of the model, except the triple interaction GTE, had a significant effect on both LERw and LERl.

As the environment has a great impact on both LERw and LERl but is not predictable before sowing (mainly because of the effect of year), we considered it as a random factor. Both producer and associate effects were then analysed by the following mixed model (model 1):


(4) Y=ijtkμ+G+iT+jE+tGT+ijGE+itTE+jtEBtk+ε(1)ijtk,

where Y_ijtk_ is the LERw or LERl obtained when the wheat genotype i was intercropped with the legume tester j in the environment t and the block k. μ is the overall mean, G_i_ is the main effect of i-*th* wheat genotype, T_j_ is the main effect of j-*th* legume tester, E_t_ is the main effect of t-*th* environment, GT_ij_ is the ij-*th* wheat genotype × legume tester interaction, GE_it_ is the it-*th* wheat genotype × environment interaction, TE_jt_ is the jt-*th* legume tester × environment interaction, EB_tk_ is the effect of k-*th* block in the t-*th* environment, and ε_ijtk_ is the ijtk-*th* residue. Wheat genotype and legume tester were regarded as fixed factors, while environment was as a random factor. With this assumption, the effects of wheat genotype (G_i_), legume tester (T_j_), and their interaction (GT_ij_) were considered as fixed, whereas all the other effects were considered as random. The random effects E_t_, EB_tk_, GE_it_, and TE_jt_, were assumed to be independently distributed with zero mean and variances σ_E_^2^, σ_EB_^2^, σ_GE(i)_^2^, and σ_TE(j)_^2^. We assumed the heteroscedasticity of the model, i.e., for the t-*th* environment, ε_ijtk_ ∼ N(0, σ_(t)_^2^). The model was fit by maximising the restricted log-likelihood with the R package nlme (version 3.1-152; Pinheiro J. et al., 2021).

### Test of Random and Fixed Terms

Each random term was tested by comparing model 1 with another one obtained by dropping the term under study (Supplementary Table 1). For instance, to test the effect of wheat genotype × environment interaction (GE_it_), model 1 was compared to the following model:


(5) Y=ijtkμ+G+iT+jE+tGT+ijTE+jtEB+tkε,ijtk

Three indicators were considered for comparison: Akaike information criterion (AIC), Bayesian information criterion (BIC), and the result of a log-likelihood-ratio test between the two models. The lower the AIC and the BIC, the better the model was, and we considered a random term as significant only if the *p*-value associated with the log-likelihood-ratio test was below 5%. Once the random terms were set, the fixed terms of the model were tested by classic analysis of variance and Fisher tests.

### Comparing Producer and Associate Effects of Wheat Genotypes When Intercropped With a Legume

The mean varietal performance of the i-*th* wheat genotype in intercropping (either producer or associate effect) is given by G_i_. The stability of this performance across the environments is given by σ_GE(i)_. When the genotypes had a significant effect on producer or associate effects, their performances were compared by pair comparisons implemented using the package emmeans (Version 1.6.1^1^). The *p*-values of the 28 possible comparisons were adjusted with the Tukey method.

### Comparing the Legume Testers for Their Ability in Classifying and Discriminating the Wheat Genotypes for Their Producer or Associate Effects

The effects of legume testers on the mean producer or associate effects were measured by T_j_ and their stability across environments by σ_TE(j)_. Therefore, these parameters gave information about the competitiveness of legumes against wheat.

If the impact of wheat genotype × legume tester interaction was significant on producer or associate effects, genotype rankings obtained with each tester were compared graphically. Particular attention was paid on pairs of genotypes that each tester was able to discriminate significantly at 5%.

## Results

### Variability of Environmental Conditions

Environmental conditions (temperature, rainfall) were close to the means recorded across the 10 previous years in the 3 locations, apart from rainfall through the growing season (21 October–20 July) in EM17, with the lowest rainfall recorded over the previous 10 years period, denoting a particularly dry season, and in DI18, with the highest rainfall recorded in the 10 previous years, denoting a particularly wet season (Table 3).

**TABLE 3:** Agronomic and environmental conditions of the 9 trials testing 8 wheat genotypes for their ability to be cropped in binary mixtures with 5 legume (pea, faba bean) tester varieties.

					Temperature (°C)							
Location *(system)*	Year	Trial/Environment	Sowing date	Harvest date	Mean	Min	Max	Rainfall (mm)- Cumulated on the growing season	Previous crop	Soil type	Soil depth	Available *N* Units (end of winter)
					
					On the growing season							
INRAE Dijon (DI)	2017	DI17	2016/10/21	2017/07/12	9.4	–9.6	36.2	460	Spring oat	clay-loam to clay	moderately deep	107 U
*(very low input)*	2018	DI18	2017/10/24	2018/07/09	10.0	–11.6	34.1	740	Spring oat	clay-loam to clay	moderately deep	31 U
	2019	DI19	2018/10/24	2019/07/17	9.9	–6.0	37.6	420	Spring oat	clay-loam to clay	moderately deep	

INRAE Estrées-	2017	EM17	2016/10/21	2017/07/18	9.4	–6.2	34.4	300	Wheat	loamy	moderately deep	117 U
Mons (EM)	2018	EM18	2017/10/27	2018/07/15	9.8	–8.6	31.7	540	Wheat	loamy	moderately deep	23 U
*(very low input)*	2019	EM19	2018/10/25	2019/07/17	9.5	–4.5	33.3	430	Wheat	loamy	moderately deep	66 U

INRAE Rennes (RE)	2017	RE17	2016/11/09	2017/07/21	10.6	–7.4	35.1	430	Grassland	loamy, beating	deep	61 U
*(organic farming)*	2018	RE18	2017/10/31	2018/07/18	10.9	–7.2	31.2	530	Maize	loamy, beating	deep	64 U
	2019	RE19	2018/11/06	2019/07/17	10.8	–3.6	35.7	430	Maize	loamy, beating	deep	74 U

At all three sites, the years 2017 and 2019 were characterised by rather dry and cold winters, followed by warm springs; 2018 presented low temperatures in February, very wet winter and spring (Supplementary Figure 1).

The storm Miguel passed through the RE19 trial in June, causing early and heavy lodging of almost all the sole peas and intercropped wheat-pea combinations.

The previous crops were straw cereals in Dijon and Estrées-Mons every year, and maize or grassland in Rennes depending on the year.

The soil types were clay-loam to heavy clay in Dijon, and loamy in Estrées-Mons and Rennes. The nitrogen available in the soil at the end of winter was 70 U N ha^–1^ on average in the nine environments, ranging from 23 U N ha^–1^ in EM18 (completed with 30 units of nitrogen at the end of March) to 117 U N ha^–1^ in EM17.

The delay between sowing and emergence varied between environments and species: from 13 to 19 days for wheat, 15 to 31 days for pea, and 15 to 37 days for faba bean, with a maximum delay of 6 days between genotypes of the same species when considering a single environment. Depending on the environment, pea emerged 2–12 days and faba bean 2–18 days after wheat.

### Wheat and Grain Legume Yields in Sole Crop and Intercrop

Yields were highly variable between environments. Wheat yields in SC, averaging 45 q/ha over the 8 genotypes and the 9 environments, ranged from 23 q/ha in DI18 to 61 q/ha in EM17 and RE19 (Supplementary Figure 2A). This corresponds to average to high yields for wheat sole crops in very low input or organic farming systems, where between 20 and 30 q/ha are usually expected^2^. On average, over the 3 years, Dijon showed the lowest SC wheat yield potential (34 q/ha), systematically lower than the average, while Rennes and Estrées-Mons showed higher yield potentials, each close to 50 q/ha. Average wheat yields across environments for genotypes with high yield potential in SC were, as expected, higher (+10 q/ha) than those of genotypes with lower potential (Supplementary Figure 2A). The average wheat yield obtained in IC across environments was 26 q/ha, with EM showing better wheat yield potential over the 3 years (36 q/ha) when compared to Dijon and Rennes, which had lower wheat yield potential (20 and 22 q/ha, respectively).

Pea yields in SC over the 3 testers averaged 36 q/ha across environments (Supplementary Figure 2B), and ranged from 14 q/ha in RE18 to 54 q/ha in DI17 and EM19, with, globally, higher pea yield potentials in Dijon and Estrées-Mons (41 and 48 q/ha, respectively) than in Rennes (20 q/ha). The average pea yield obtained in IC across environments was 24 q/ha with, globally, the same pea yield potential over the 3 years at the 3 locations (±1 q/ha).

Faba bean yields in SC over the 2 testers varied from 25 q/ha in RE18 to 43 q/ha in EM19. The average faba bean yield in SC over the 8 environments was 33 q/ha with similar faba bean yield potential over the 3 years for the 3 locations (±2 q/ha). The average faba bean yield obtained in IC across environments was 21 q/ha. Dijon and Rennes showed better yield potential over the 3 years (24 and 25 q/ha, respectively) when compared to Estrées-Mons, which had lower yield potential (16 q/ha).

### Partial Land Equivalent Ratios

**FIGURE 2:**
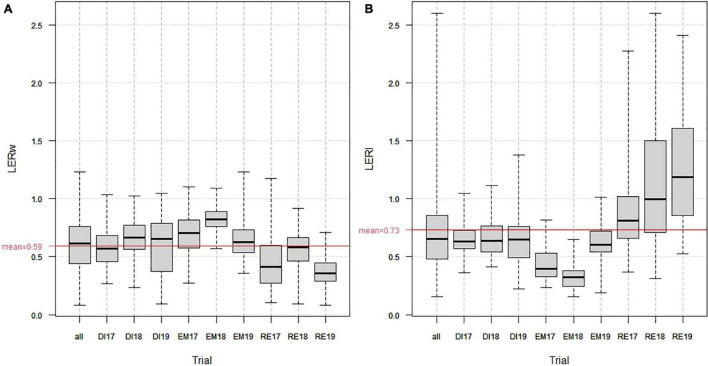
Distribution of partial Wheat **(A)** and Legume **(B)** Land Equivalent Ratios over the 9 environments (all) and for each environment (DI, Dijon; EM, Estrées-Mons; RE, Rennes; 17, 2016/2017 trial; 18, 2017/2018 trial; 19, 2018/2019 trial). The red bars show the LERw **(A)** and LERw **(B)** means across all environments.

Across all environments and intercrops, LERw varied between 0.08 and 1.23 and LERl between 0.15 and 2.6 (Figure 2), with high variations between environments. In mean, Estrées-Mons showed the highest LERw, comprised between 0.64 in 2019 and 0.82 in 2018 (Figure 2A), together with the lowest LERl (Figure 2B). Dijon, where wheat yields were lowest both in SC and in IC, showed medium LERw, presumably stable between years, ranging from 0.54 in 2017 to 0.65 in 2018, together with medium, and, presumably stable between years, LERl. Rennes, with high wheat yields in SC and low wheat yields in IC, showed lowest LERw (from 0.37 in 2019, linked to high lodging in IC, to 0.56 in 2018), together with high and very variable LERl (due to very low pea yields obtained in SC over the 3 years), sometimes over 1 (meaning that in some cases, the legume yields obtained in IC were higher than those obtained in SC).

### Impact of Environment, Wheat Genotype, and Legume Tester on Wheat Mixing Ability

Partial land equivalent ratios across wheat genotypes, environments, and testers averaged 0.59 for LERw and 0.73 for LERl (Figure 2). The estimates of standard deviations and confidence intervals of LERw (SD = 0.04, CI = 0.51–0.68) and LERl (SD = 0.1, CI = 0.53–0.93) showed that if LERl was, on average, higher than LERw, it was also less precisely estimated.

The observed LERw and LERl were highly dependent on trial. Indeed, environmental effects on both variables were significant at 0.1% (Table 4), with standard deviations of 0.1159 and 0.2933 for the models on LERw and LERl, respectively (Table 5). Therefore, tester legume varieties were subjected to 2.5 times higher variations of their partial LER between environments than wheat genotypes.

**TABLE 4:** *p*-Values of log-likelihood-ratio tests between reference and test models (see Supplementary Table 1) compared to assess the relevance of environment (E), block (EB), wheat genotype × environment interaction (GE), and legume tester variety × environment interaction (TE) random effects on partial wheat genotypes and legume tester varieties land equivalent ratios (LERs), and the relevance of estimating residual variance by environment [σ_(t)_^2^], GE variance by genotype [σ_GE(i)_^2^], and TE variance by legume tester [σ_TE(j)_^2^].

Term	*p*-value LERw	*p*-value LERl
E	<10^–16^	<10^–16^
EB	3.4 × 10^–12^	2.0 × 10^–8^
GE	<10^–16^	0.0043
TE	<10^–16^	<10^–16^
σ**_(t)_**^2^	<10^–16^	<10^–16^
σ_GE_**_(i)_**^2^	0.9317	0.7112
σ_TE_**_(j)_**^2^	0.0193	0.3885

**TABLE 5:** Standard deviations estimated for residues on random terms [environment (E), wheat genotype × environment interaction (GE), legume tester variety × environment interaction (TE), and block (EB)].

Term	LERw	LERl
E	0.1159	0.2933
EB	0.0392	0.0493
GE	0.0498	0.0270
TE (Fresnel)	0.0640	
TE (Geronimo)	0.0601	
TE (Irena)	0.1600	
TE (Olan)	0.1480	
TE (Spencer)	0.0006	0.1723
ε	0.1104	0.0987

*As TE variance differed significantly in legume tester only for LERw, standard deviation of the TE term was estimated by legume tester only for the model on LERw.*

Environment also significantly influenced the effects of wheat genotypes and legume testers on both LERw and LERl (*p*-values of GE and TE terms below 0.01 as shown in Table 4). However, the impact of environment on wheat genotype effect remained moderate on both variables, with standard deviations of the GE term below 0.05 (Table 5), whereas it was much higher on legume testers effect, with standard deviations of the TE term over 0.15 on LERl, and for faba bean testers on LERw (Table 5).

Mean residual standard deviations of the models on LERw and LERl, i.e., 0.1104 and 0.0987, respectively (Table 5), showed that experimental variance remained significant compared to other sources of variability. Furthermore, this experimental variance clearly differed from one environment to another (*p*-values < 0.001 as shown in Table 4). The residual standard deviations indeed ranged from 0.0774 in RE19 to 0.198 in RE17 for the model on LERw and from 0.0824 in EM18 to 0.3818 in RE18 for the model on LERl, which indicates that some trials were more precise than the others.

Wheat genotype had a significant effect on both LERw and LERl (*p*-values of G term below 0.001 as shown in Table 6), meaning that some wheat genotypes are likely to have better producer and/or associate effects on intercropping than others. As the standard deviation of GE interaction did not depend on the genotype under study (*p*-value > 0.7 for LERw and LERl as shown in Table 4), we were not able to identify wheat genotypes whose effect on LERw or LERl was more stable between environments than others.

**TABLE 6:** *p*-Values of Fisher test on wheat genotype (G), legume tester variety (T) main effects and their interaction (GT) from an ANOVA analysis.

Term	LERw	LERl
G	3.9 × 10^–5^	3.5 × 10^–13^
T	0.0023	0.0331
GT	0.0296	0.1069

Legume tester also had a significant effect on both LERw and LERl (*p*-values of T term below 0.05 as shown in Table 6). The effect of TE interaction on LERw depended on legume tester (*p*-value < 0.05 as shown in Table 4). Therefore, the effects of the pea tester varieties (Spencer, Fresnel, and Geronimo, 0.0001 < sd < 0.065 as shown in Table 5) were at least twice more stable between environments than the effects of faba bean testers (Irena and Olan, 0.148 < sd < 0.16 as shown in Table 5). This difference in stability between legume testers was not observed for LERl (*p*-values > 0.38 as shown in Table 4), with all the testers’ effects being strongly impacted by environments in this case (SD = 0.172 as shown in Table 5).

Finally, the interaction between wheat genotype and legume tester variety had a significant effect on LERw (*p*-value of GT term below 0.05 as shown in Table 6) and a less but still likely significant effect on LERl (*p*-value = 0.1069 as shown in Table 6). Thus, it appeared that the producer or associate effect of a wheat genotype on intercropping may depend on legume tester, making the choice of this tester a key issue both in screening and in breeding.

### Classification of Wheat Genotypes Based on Their Average Producer and Associate Effects

On average, over the 9 environments and 5 legume testers, LERw estimates ranged from 0.52 for Forcali/Rebelde to 0.67 for Ehogold, with an average of 0.59; and LERl estimates ranged from 0.67 for Flamenko to 0.82 for Forcali/Rebelde, with an average of 0.73 (Table 7). LERt ranged from 1.26 (for Flamenko) to 1.36 (for Ehogold and CF14336), with the five other genotypes having a LERt close to the 1.32 mean.

**TABLE 7:** Mean partial and total land equivalent ratios (LER) across 9 environments for the 8 wheat genotypes intercropped with the 5 legume testers.

	LERw		LERl		LERt

Mean (μ)	0.59		0.73		1.32
**Wheat genotype**					
Ehogold	0.67	a	0.69	cd	1.36
Attlass	0.61	ab	0.70	cd	1.31
Renan	0.60	abc	0.72	bcd	1.32
Flamenko	0.59	abc	0.67	d	1.26
RE13003	0.59	abc	0.73	bcd	1.32
CF14336	0.59	abc	0.77	ab	1.36
Geny	0.57	bc	0.75	bc	1.32
Forc-Reb	0.52	c	0.82	a	1.34
**Legume tester**					
Spencer	0.65	a	0.80	ab	1.45
Fresnel	0.65	a	0.71	ab	1.35
Geronimo	0.62	a	0.83	a	1.45
Irena	0.59	ab	0.57	b	1.16
Olan	0.45	b	0.75	ab	1.21

*LERw, partial wheat LER; LERl, partial legume LER; LERt, total LER. Genotypes with the same letter are not significantly different at the 5% threshold.*

Among the 28 possible comparable pairs of wheat genotypes, only 3 showed significant differences in their producer effect (LERw) across all legume testers at 5%, i.e., Ehogold vs. Geny, Ehogold vs. Forcali/Rebelde, and Attlass vs. Forcali/Rebelde (Table 8A). Renan, Flamenko, RE13003, and CF14336 formed a homogeneous group, with an LERw very close to the 0.59 average across legume testers (Table 7). A larger set (10) of pairs of wheat genotypes showed significant differences in their associate effect (LERl) at 5%, i.e., Forcali/Rebelde vs. all the other genotypes except for CF14336, CF14336 vs. Attlass, Ehogold, and Flamenko, and Geny vs. Flamenko (Table 8B).

**TABLE 8:** Significant differences between pairs of wheat genotypes intercropped with legume tester varieties for their **(A)** producer and **(B)** associate effects on average over all testers and for each of the 5 testers studied.

A - LERw		Legume testers varieties					
		Mean	Fresnel	Geronimo	Spencer	Olan	Irena

Wheat genotype 1	Wheat genotype 2						
Ehogold	Flamenko					(+)	

Ehogold	RE13003			++	(+)		

Ehogold	CF14336	(+)					

Ehogold	Geny	++		+++	+		

Ehogold	Forc-Reb	+++	+	+++	+++	+++	(+)

Attlass	Forc-Reb	+	+		+		

Renan	Forc-Reb	(+)					

Flamenko	Forc-Reb				(+)		

Geny	Forc-Reb					(+)	

**B - LERl**		**Legume testers varieties**					
		**Mean**	**Fresnel**	**Geronimo**	**Spencer**	**Olan**	**Irena**

**Wheat genotype 1**	**Wheat genotype 2**						

Forc-Reb	Geny	+		+			

Forc-Reb	RE13003	+++		+++			

Forc-Reb	Renan	+++		+++	+		

Forc-Reb	Attlass	+++	+	+++	+++		

Forc-Reb	Ehogold	+++	++	+++	+++		

Forc-Reb	Flamenko	+++		+++	+++	+	
CF14336	Renan			(+)			

CF14336	Attlass	+			(+)		

CF14336	Ehogold	++	(+)	+	+		

CF14336	Flamenko	+++		+++	++		

Geny	Flamenko	+		++	+		

*The wheat genotypes are ranked from top to bottom according to decreasing LER in question. Pairs showing no significant difference are not shown. +++, p-value < 0.001 and genotype 1 > genotype 2. ++, p-value < 0.01 and genotype 1 > genotype 2. +, p-value < 0.05 and genotype 1 > genotype 2. (+), p-value < 0.1 and genotype 1 > genotype 2.*

**FIGURE 3:**
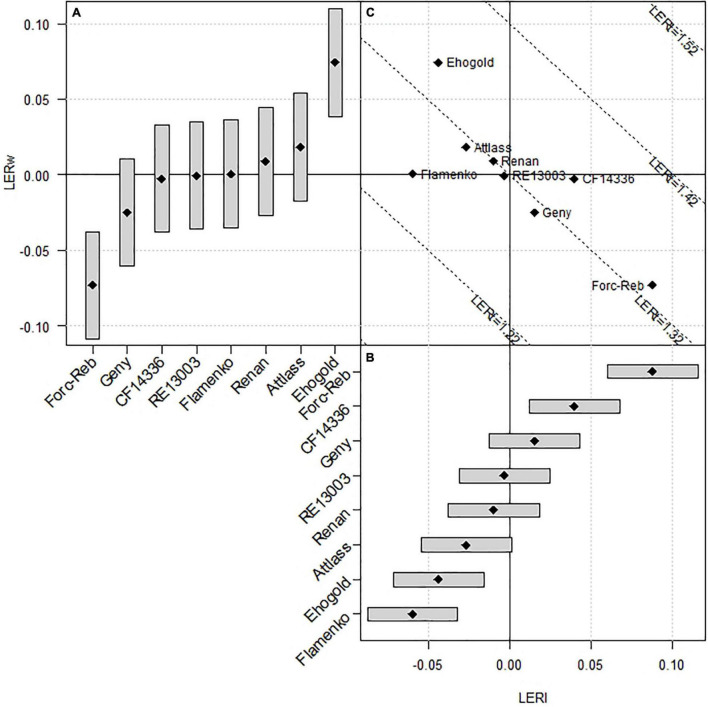
Effects of the 8 wheat genotypes on LERw (Producer effect, graph A) and LERl (Associate effect, graph B). The dashed lines in graph (C) have the same total LER (LERt). Average LERt is 1.32 as the sum of average LERw (0.59) and average LERl (0.73).

Each wheat genotype can, thus, be characterised by its effect on both LERw (producer effect) and LERl (associate effect). Both effects appear to be negatively linked, with a slope not significantly different from –1, meaning that improving the producer effect generally leads to a similar decrease in the associate effect (Figure 3). The wheat genotypes Attlass, Renan, RE13003, Geny, and Forcali/Rebelde are all located very close to the 1.32 LERt mean, equal to a 1.32 mean but with different LERw and LERl contributions to LERt. The 3 other genotypes deviate from this mean line, with Flamenko having the lowest LERl and average LERw, thus leading to lowest LERt; Ehogold showing the highest LERw but a rather low LERl, and CF14336 showing a high LERl together with an average LERw, both leading to highest LERts.

The correlation coefficients between genotypes’ producer or associate effects and their yields, height or lateness at heading stage estimated in SC (Table 1) are presented in Table 9. Producer effects (LERw) were positively and significantly correlated to lateness and height at the heading stage but had a low correlation to yield. Height, lateness and yield were all negatively correlated to associate effects (LERl) but the correlations were not significant at 15%. These results must be considered cautiously as the number of wheat genotypes under study was small and the correlations were influenced by some genotypes like Ehogold and Forcali/Rebelde that are among the genotypes presenting the lowest and highest producer and associate effects.

**TABLE 9:** Estimates and *p*-values (pv) of Pearson correlation coefficients between lateness at heading stage, height at heading stage and yield in SC, and producer (LERw) and associate (LERl) effects of the 8 wheat genotypes.

	LERw	LERl
Lateness at heading stage (SC)	0.67 (pv = 0.07)	–0.45 (pv = 0.26)
Height at heading stage (SC)	0.73 (pv = 0.04)	–0.34 (pv = 0.40)
Yield (SC)	0.15 (pv = 0.73)	–0.54 (pv = 0.17)

### Legume Testers Competitiveness and Ability to Discriminate Wheat Genotypes

On average, over the nine environments and across the eight wheat genotypes, Olan was the most competitive tester with a LERw of 0.45, whereas Spencer, Fresnel, and Geronimo were the least, showing the highest LERw of 0.65, 0.65, and 0.62, respectively (Table 7).

**FIGURE 4:**
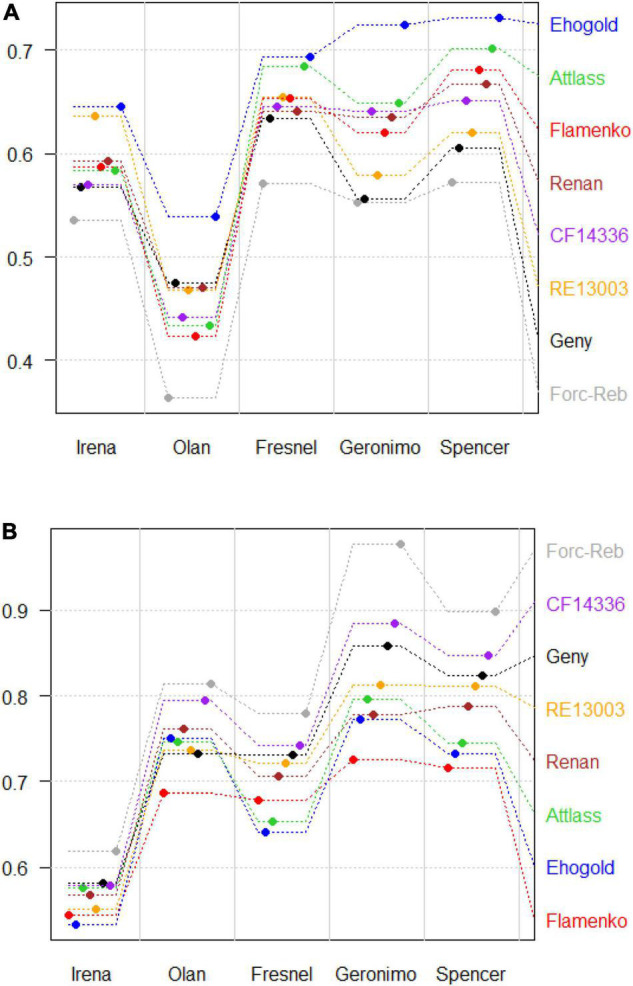
Mean partial **(A)** wheat (LERw) and **(B)** legume (LERl) land equivalent ratios across environments for 8 wheat genotypes intercropped with 5 legume testers. The wheat genotypes are ranked from left to right for each tester according to their average LER across all the 5 testers.

Wheat genotype × legume tester interaction was significant for LERw (*p*-value < 0.05 as shown in Table 6) and less significant but still possible for LERl (*p*-value = 0.11 as shown in Table 6). This type of interaction may be qualitative (inversion in wheat genotype classification between legume testers) or quantitative (without modification of wheat genotypes classification), or both. Depending on the tester considered, the wheat genotypes were not always classified in the same order, either for LERw or LERl, indicating possible qualitative interactions (Figure 4). On all the testers, 9 and 11 pairs of wheat genotypes among the 28 possible combinations showed significant differences in their LERw (Table 8A) and LERl (Table 8B). When classification inversions occurred between testers, they did not give rise to significant differences between wheat genotypes. Indeed, when a significant difference was observed between two genotypes for one tester, they were either ranked the same way or not significantly different when intercropped with the other testers. Therefore, wheat genotype × legume tester interaction was mainly quantitative.

The ability of a legume tester to discriminate wheat genotypes for their producer (LERw) and associate (LERl) effects can be approached graphically or by the number of significantly different pairs of genotypes it can dissociate. All the legume testers did not seem to have the same discrimination potential for LERw and/or LERl (Figure 4). Individual tester ability to distinguish wheat genotype pairs differed greatly between testers (Table 8): at a 5% threshold, Irena did not distinguish any pair of genotypes both for LERw and LERl, Olan only distinguished the most different pairs (Ehogold vs. Focali/Rebelde for LERw and Forcali/Rebelde vs. Flamenko for LERl), and Fresnel distinguished only two pairs for both LERw and LERl. Interestingly Spencer and Geronimo distinguished the highest number of wheat genotype pairs for both LERw (3 and 3, respectively) and LERl (7 and 9, respectively).

## Discussion

### Approaches to Varietal Mixing Ability Assessment

Studying the impact of varieties and species on the performance of binary intercropping raises a methodological problem, since all possible combinations (m genotypes of the target species A × n genotypes of tester B) cannot be considered in all environments. The experimental effort in identifying varietal mixing abilities may be reduced by (i) testing genotypes with contrasting traits, assuming these traits are likely to impact performance (Hauggaard-Nielsen and Jensen, 2001; Baxevanos et al., 2017), and mixing them with chosen tester representatives of the possible mixed species and genotypes, (ii) reducing experimental investment using incomplete designs, making it possible to maintain sufficient precision and statistical power to identify differences between genotypes (Haug et al., 2021), or (iii) coupling experimental designs with agronomic modelling (Gaudio et al., 2019).

We chose the first strategy, assuming that the producer and associate effects of a species involved in intercropping could depend on choices of both genotypes in the species and the mixed tester species or variety. Our results on wheat-grain legume intercrops confirmed this hypothesis by showing that (1) contrasting wheat genotypes have different producer and associate effects and (2) the choice of grain legume tester has an important impact on our ability to differentiate wheat varieties for their producer and associate effects.

### A Wide Range of Environments Strengthen the Robustness of the Results

The yield potential of each species in sole crop or intercropping can be significantly impacted by the genotype under study, genotype of the mixed crop, cropping practices such as sowing densities, and by intercrop pedo-climatic conditions, i.e., environments. Indeed, all factors directly or indirectly impact the so-called 4Cs (Justes et al., 2021), mainly competition and complementarity. Therefore, the robustness and genericity of the resulting relative contributions of each intercropped species to yield highly depend on the range of environments under study. The multi-environment trial network described in this report indeed integrated both conventional (DI and EM) and organic (RE) cropping conditions and a large range of environments, including extreme and unusual ones. EM17 presented a lack of rainfall throughout the season, RE19 experienced an important storm that resulted in high and variable rate of lodging for all SC and IC pea plots, and DI18 presented particularly high rainfall and low levels of N by the end of winter, which clearly altered wheat and legume tester yields (Supplementary Figure 2). As a whole, both LERw and LERl differed significantly between environments. The relative contributions of these partial LERs to total LER differed greatly between environments, showing that the chosen environments cover a large range of biotic and abiotic stress patterns and competition situations between mixed species/varieties, some conducive for both species and some conducive for one of the species and not the other.

### Sowing Densities for Balanced Mixtures

Relative seeding rates in mixtures are considered an important parameter in the performance of mixtures (Neumann et al., 2007; Barker and Dennett, 2013; Pötzsch et al., 2019). They influence the competition between species throughout the crop cycle and can thus impact the estimation of the producer and associate effects of tested wheat varieties. For practical reasons (number of microplots per trial), we could not test several relative seeding densities per type of mixture and chose densities allowing balanced production of each species. Indeed, balanced mixtures allow to discriminate between varieties on both their capacity to produce and to make produce. The objective of balanced production between wheat and legume was globally reached with partial LERs generally higher than 0.5 and similar for each species. However, it appears that the partial LERs of Hr peas are higher than those of wheat, and this would probably have justified lowering slightly their seeding rate in IC compared to the sole crop. The partial LERs of the Olan tester are significantly higher than those of the associated wheat, arguing for a significant reduction in the seeding rate of faba bean in IC for this tester. Finally, we cannot exclude that the ability to discriminate between wheat varieties would be different with very unbalanced seeding rates of the intercropped species.

### Partial Land Equivalent Ratios Are Adapted to Identify Genotypes Adapted to Intercrop

Many indices may be used to assess species interactions in intercrops for growth and/or yield, including ratios (such as partial and total LERs), simple differences in performances (Haug et al., 2021), or even differences between ratios (aggressivity). Some indices (such as relative efficiency index or comparative absolute growth rate) take into account the dynamics of competitive interactions in growth. Others finally tend to separate interspecific from intraspecific interactions (reviewed in Bedoussac and Justes, 2011). Since our purpose was to assess the final relative yield outcome of the interaction rather than compare relative growth dynamics or analyse separately intraspecific from interspecific interactions, it looked sound to compare wheat varieties for their producer and associate effects based mainly on their partial LERs for yield. Indeed, this index that allows to quantify mixture productivity compared to the sole crops was acknowledged as relevant and versatile (Bedoussac and Justes, 2011), and it was widely used and adapted for large meta-analyses (reviewed in van Der Werf et al., 2021). The choice to use yield ratios (partial LERs) rather than yield differences to compare the mixing abilities of wheat genotypes was also supported by their fixed sowing density in IC, set as a percentage of their sowing density in SC. Therefore, we expected that their yield in IC would also depend on their yield potential in SC, which may vary from one environment to another, and should be expressed as a percentage of the yield in SC rather than through a yield loss between SC and IC. The chosen wheat genotypes also differed significantly in terms of yield potentials in SC (for instance, the Flamenko and Ehogold genotypes presented a mean yield of 50 and 40 q/ha in SC, respectively), so using the yield loss between SC and IC to qualify mixing ability would have suffered a possible confusion with productivity. The low and non-significant correlation coefficients between LERw and yield in SC for the 8 wheat genotypes under study (Table 9) validate this choice *a posteriori*. Finally, the application of models (this issue) to differences led to much higher G × E interactions (data not shown). Using yield ratio instead of yield loss between IC and SC, therefore, makes it possible to differentiate the mixing ability of varieties from their productivity in SC and to compare properly the varieties for their mixing ability in very different environments.

We did not correct partial LERs for initial sowing densities in IC, because the sowing ratios were all identical between wheat genotypes for a given mixture and chosen according to a potential farmer’s objective aiming at harvesting a balanced quantity of both species.

### Wheat Genotypes Show Contrasted Profiles of Mixing Ability

The observed significant differences between wheat genotypes in their ability to produce (producer effect) and make their mixed tester produce (associate effect) in intercrop confirm the relevance of the traits considered in the choice of wheat varieties (i.e., potential yield, earliness, and height at heading stage in SC). Indeed, the traits were previously reported to impact significantly the competitive ability of a species in general (Annicchiarico et al., 2019), and particularly of cereals (Haug et al., 2021; Kammoun et al., 2021) in IC. Considering both productive and associate effects of the genotypes, it is likely that complementation for resource use took place in most situations, since mean LERt values across environments were all above 1.26 (Figure 3), which confirms a large consensus from previous results on intercropping binary mixtures (Bedoussac et al., 2015; Stomph et al., 2020). In most cases, competition between species also took place, since reduction in the partial LER of one species is generally compensated by a rise in the partial LER of the mixed species. Therefore, while some genotypes showed higher producer or associate effects, they were hardly different in terms of their global mixing ability (producer + associate effects). Noticeable exceptions were observed for the wheat genotype Flamenko, whose competitiveness does not let the legume produce at an expected level, and wheat genotypes Ehogold and CF14336, which generated higher mean LERt than the others (Figure 3) probably because of stronger facilitation effects.

Using the mean values of yield, earliness and height of each wheat genotype in SC, we have identified a possible impact of earliness or height on producer effects; late and high genotypes showing higher LERw. However, these relationships are particularly influenced in our study by the behaviour of the Ehogold and Forcali/Rebelde genotypes and should be confirmed on a larger set of genotypes. No other obvious links between these traits and producer or associate effects were detected. This can be explained both by the small number of genotypes under study that does not allow for a clear relationship to be established, and by other plant and canopy traits that may impact competition and facilitation between species in IC. Indeed, in addition to height, earliness, and productivity, plant and canopy traits likely to be involved are numerous, such as early vigour, light interception, leaf area index (LAI), tillering ability, canopy architecture, crop ground cover, nutrient use efficiency, lodging, and disease resistance. Canopy height, lodging, and maturity date were, for instance, shown to be important determinants of forage yield and quality when oat was intercropped with vetch species (Assefa and Ledin, 2001). Furthermore, these traits interact strongly with cropping management, so their expression in SC is likely not to predict their expression in IC (Moutier et al., 2018, 2021; Kammoun et al., 2021). Indeed, the plasticity in traits initially identified in SC is a key issue to understanding cultivar adaptation to IC (Gaudio et al., 2019). Therefore, a study is in progress to define the plasticity of varietal and canopy traits in IC, test a larger range of variations of these traits, and try to identify other traits possibly correlated that may impact competitive ability in a complex way. It is likely, for instance, that different dynamics of canopy closure in IC among Ehogold, Flamenko, Forcali/Rebelde, and CF14336 may explain a part of their significant different competitiveness schemes (data not shown).

### Grain Legume Tester Varieties Differ in Their Ability to Discriminate Wheat Genotypes

A significant impact of a mixed species on the performance of a target species has often been shown (Wendling et al., 2017). On the contrary, a recent study focusing on the general mixing (GMA) and specific mixing (SMA) abilities of barley genotypes showed that SMA is very low compared to GMA, which led to the conclusion that the performance of a genotype in intercropping hardly depends on mixed tester genotype, and that tester choice is not a key issue (Haug et al., 2021). Our study partially confirms these results, as there are no significant differences in the ranking of wheat genotypes according to their mixing abilities when the genotypes were intercropped with different grain legume testers. However, we report that the grain legume testers have different abilities to discriminate wheat genotypes, with some having a more stable impact on LERw than the others.

Among the grain legume testers, Geronimo and Spencer (Hr pea varieties) significantly discriminate the largest number of wheat genotype pairs for their mixing ability (both for producer and associate effects; Table 8), and they allow the wheat genotypes both to produce and to maintain associate effects, leading to highest LERt (Table 7). Fresnel (hr pea variety) only discriminates wheat genotypes that are extreme for their LERw and/or LERl (Table 8), and may be slightly less competitive than Geronimo and Spencer (not significant; Table 7). The faba bean grain legume testers Olan and Irena both fail in discriminating wheat genotype pairs for their mixing ability (except Olan with Forcali/Rebelde vs. Ehogold for LERw and Forcali/Rebelde vs. Flamenko for LERl; Table 8). They also display lower LERw (although this effect is not stable between environments), leading to lower LERt, showing better competitiveness towards wheat (Table 7). Olan is the most competitive, probably because of its height and soil coverage power (Table 1), while Irena is less competitive (it is shorter and covers the soil more slowly; Table 1).

The number of representatives in each of the three cultivated types (pea Hr, pea hr, and faba bean) is far from being enough to definitively make a conclusion on potential interest on them as testers. Differences between Geronimo and Spencer on one side, and between Olan and Irena on the other side, show that there may be a variation in the cultivated types. We can, however, hypothesise that their different development dynamic cycles known from sole cropping probably affect differently their competitiveness. For the Hr pea testers, weak development at early stages allows for the wheat to establish during the winter, and then strong development from ramifications at the end of the cycle makes it possible to build up their own production (Lejeune-Hénaut et al., 2008). Stronger development during the whole cycle for hr pea testers or very strong development from the very early steps of the cycle during the winter for the faba bean testers do not help wheat to build up its own production. It is likely that discrepancies in both competitiveness and ability to discriminate wheat genotypes also result from traits impacting the relative use of resources (light interception and water and nutrient use from the soil due to differences in root development dynamics).

Although further confirmation may be needed, using one or two Hr pea testers, for both their competitiveness profile and their ability to select between wheat genotypes, may be the optimal way to assess and discriminate wheat genotype (or varieties) mixing abilities without checking for large sets of genotypes in the mixed species. A collateral benefit is the likely more synchronous ripening with wheat of this Hr pea than hr pea and faba bean.

### Breeding for Mixing Ability Should Consider Both Producer and Associate Effects

There is a rather large consensus stating that since the higher performing genotypes in SC are not necessarily the higher performing in IC (Francis, 1981), specific breeding programs to optimise mutual cultivar adaptation to intercropping are needed (Nelson and Robichaux, 1997; Hauggaard-Nielsen and Jensen, 2001; Annicchiarico et al., 2019; Kammoun et al., 2021). This, however, includes choice of traits to select in SC and IC on different scales (plant, canopy) and rapid and cost-effective methods for their measurements, probably the combination of SC and IC evaluation on different steps of the selection process and recurrent intercrosses in each of the mixed species for recombination steps (Wright, 1985). This study opens the way to simplifying partly this process, since the preliminary choice of a tester variety in the mixed species may reduce the number of mixtures to be tested in the selection steps and limit the recombination steps to a target species. As usual, in breeding, developing breeding programs dedicated to adaptation to binary IC depends on both the expected objective of a crop (balanced production between species or a priority on one or the other IC component, combined with a number of ecosystemic services) and whether the purpose is to create a variety that would be adapted to both SC and IC or specifically adapted to IC. We suggest that these two points are a prerequisite for defining primary screening traits, selection schemes (SC or IC at early and late generation levels), and procedures for variety certification. Our study does not definitely clarify these points but clearly confirms that both varietal choice, with some varieties moving away from the negative correlation between the producer and associate effects, and identification of an adequate tester species or variety are key points to move towards the expected objective of breeding for IC, and that, in most cases, breeding should consider both the producer and the associate effects.

## Conclusion

Wheat genotypes, therefore, show various mixing abilities. Some lose more yield (in % of their SC yield) and/or cause greater yield loss to mixed species than others when intercropped. This confirms that the varietal factor is a key issue for farmers who need to consider the mixing ability of varieties when they choose to optimise crop yields as well as potential ecosystem services.

Considering that the impact of environment on wheat genotype effect on LERw and LERl remained moderate, that some genotypes seem to stand out from the negative correlation between the producer and associate effects, and, finally, that the ability to produce in intercropping of a variety does not seem to be correlated with its SC yield potential, developing breeding methods and procedures for mixing ability seems both possible and necessary. Among these, choice of tester, which seems to have a little impact on the ranking of the mixing ability of wheat genotypes but has an impact on genotype discrimination, can be helpful to breeders to reduce the number of combinations to be tested when screening large numbers of wheat genotypes for their mixing ability. A study is currently in progress, with a view to registering wheat varieties bearing the mention “adapted to intercropping”.

## Data Availability Statement

The raw data supporting the conclusions of this article will be made available by the authors, on demand, without undue reservation.

## Author Contributions

NM, AB, and AG wrote the manuscript. NM, AB, EH, PM, and MF designed the experiments. PM, EH, and NM managed the Dijon, Estrées-Mons, and Rennes experiments, respectively. SF and AG wrote the R scripts for the analyses. All authors reviewed the final version of the manuscript.

## Conflict of Interest

MF was employed by Agri-Obtentions. The remaining authors declare that the research was conducted in the absence of any commercial or financial relationships that could be construed as a potential conflict of interest.

## Publisher’s Note

All claims expressed in this article are solely those of the authors and do not necessarily represent those of their affiliated organizations, or those of the publisher, the editors and the reviewers. Any product that may be evaluated in this article, or claim that may be made by its manufacturer, is not guaranteed or endorsed by the publisher.

## Footnotes

1
https://declaredesign.org/r/estimatr/articles/emmeans-examples.html?msclkid=f0d2191fc0f311ecb250231c4530e485

2
https://www.agencebio.org/vos-outils/les-chiffres-cles/
