# Supplementary material for: Mixing Ability of Intercropped Wheat Varieties: Stability Across Environments and Tester Legume Species
Source: Front Plant Sci. 2022 Jun 9;13:877791. doi: 10.3389/fpls.2022.877791 (PMC9218859; doi:10.3389/fpls.2022.877791)
Supplement: Supplementary file 1 [file Data_Sheet_1.docx]

**Supplementary Table 1. Reference and test models compared to test the significance of environment (E), block (EB), wheat genotype x environment interaction (GE) and legume tester variety x environment interaction (TE) random effects on partial wheat genotypes and legume tester varieties LERs, and the relevance of estimating residual variance by environment (σ_(t)_^2^), GE variance by genotype (σ_GE(i)_^2^) and TE variance by legume tester (σ_TE(j)_^2^)**

| Term | reference model | test model |
| --- | --- | --- |
| σ**_(t)_**^2^ | Y_ijtk_ = µ + G_i_ + T_j_ + E_t_ + GT_ij_ + GE_it_ + TE_jt_ + EB_tk_ + ε_ijtk_ with ε_ijtk_ ~ N(0, σ_(t)_^2^) | Y_ijtk_ = µ + G_i_ + T_j_ + E_t_ + GT_ij_ + GE_it_ + TE_jt_ + EB_tk_ + ε_ijtk_ with ε_ijtk_ ~ N(0, σ^2^) |
| σ_GE_**_(i)_**^2^ | Y_ijtk_ = µ + G_i_ + T_j_ + E_t_ + GT_ij_ + GE_it_ + TE_jt_ + EB_tk_ + ε_ijtk_ with GE_it_ ~ N(0, σ_GE(i)_^2^) | Y_ijtk_ = µ + G_i_ + T_j_ + E_t_ + GT_ij_ + GE_it_ + TE_jt_ + EB_tk_ + ε_ijtk_ with GE_it_ ~ N(0, σ_GE_^2^) |
| σ_TE_**_(j)_**^2^ | Y_ijtk_ = µ + G_i_ + T_j_ + E_t_ + GT_ij_ + GE_it_ + TE_jt_ + EB_tk_ + ε_ijtk_ with TE_jt_ ~ N(0, σ_TE(j)_^2^) | Y_ijtk_ = µ + G_i_ + T_j_ + E_t_ + GT_ij_ + GE_it_ + TE_jt_ + EB_tk_ + ε_ijtk_ with TE_jt_ ~ N(0, σ_TE_^2^) |
| EB | Y_ijtk_ = µ + G_i_ + T_j_ + E_t_ + GT_ij_ + GE_it_ + TE_jt_ + EB_tk_ + ε_ijtk_ | Y_ijtk_ = µ + G_i_ + T_j_ + E_t_ + GT_ij_ + GE_it_ + TE_jt_ + ε_ijtk_ |
| GE | Y_ijtk_ = µ + G_i_ + T_j_ + E_t_ + GT_ij_ + GE_it_ + TE_jt_ + EB_tk_ + ε_ijtk_ | Y_ijtk_ = µ + G_i_ + T_j_ + E_t_ + GT_ij_ + TE_jt_ + EB_tk_ + ε_ijtk_ |
| TE | Y_ijtk_ = µ + G_i_ + T_j_ + E_t_ + GT_ij_ + GE_it_ + TE_jt_ + EB_tk_ + ε_ijtk_ | Y_ijtk_ = µ + G_i_ + T_j_ + E_t_ + GT_ij_ + GE_it_ + EB_tk_ + ε_ijtk_ |
| E | Y_ijtk_ = µ + G_i_ + T_j_ + E_t_ + GT_ij_ + ε_ijtk_ | Y_ijtk_ = µ + G_i_ + T_j_+ GT_ij_ + ε_ijtk_ |


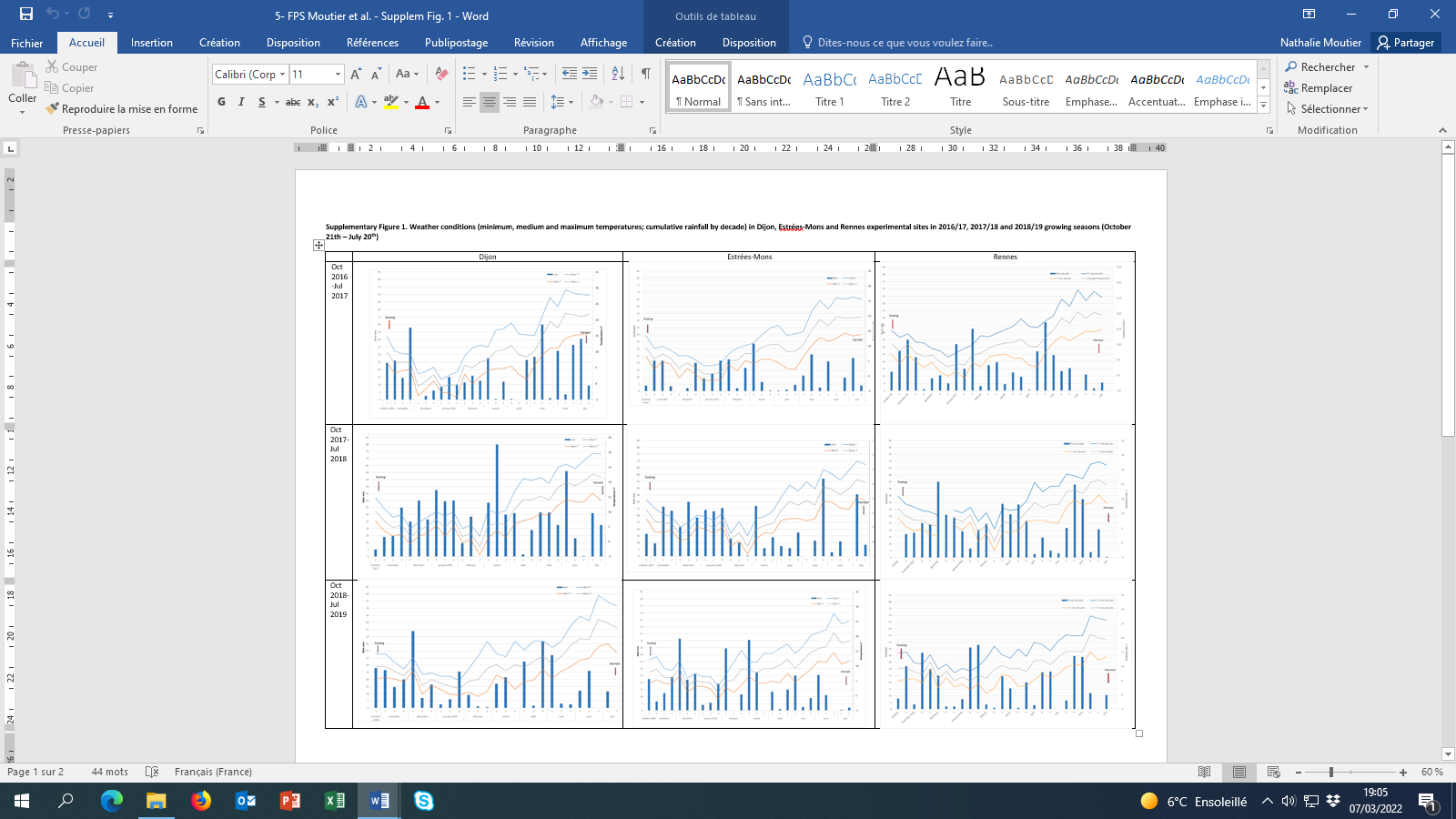


**Supplementary Figure 1. Weather conditions (minimum, medium and maximum temperatures; cumulative rainfall by decade) in Dijon, Estrées-Mons and Rennes experimental sites in 2016/17, 2017/18 and 2018/19 growing seasons (October 21th – July 20^th^)**

| 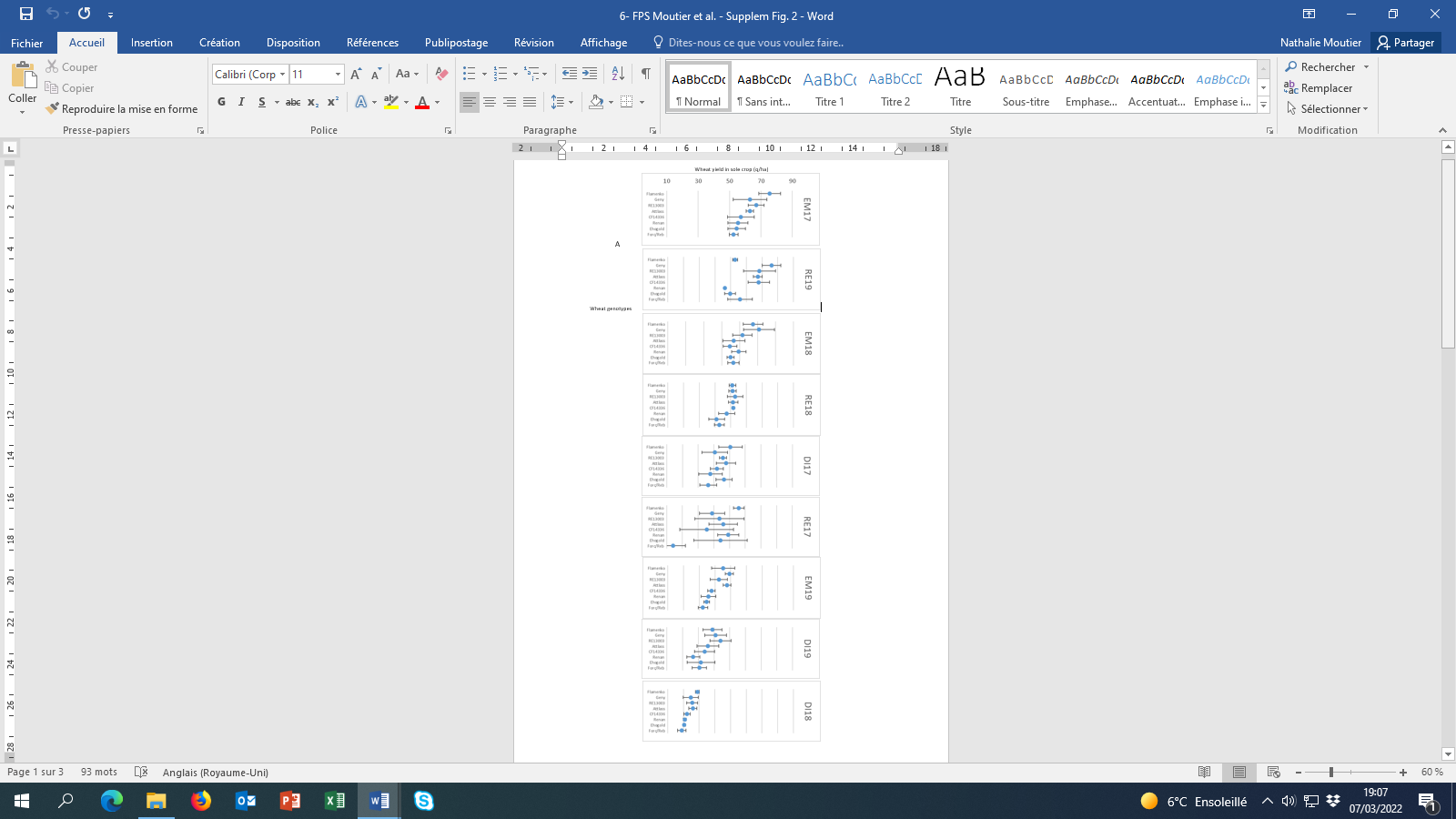 | 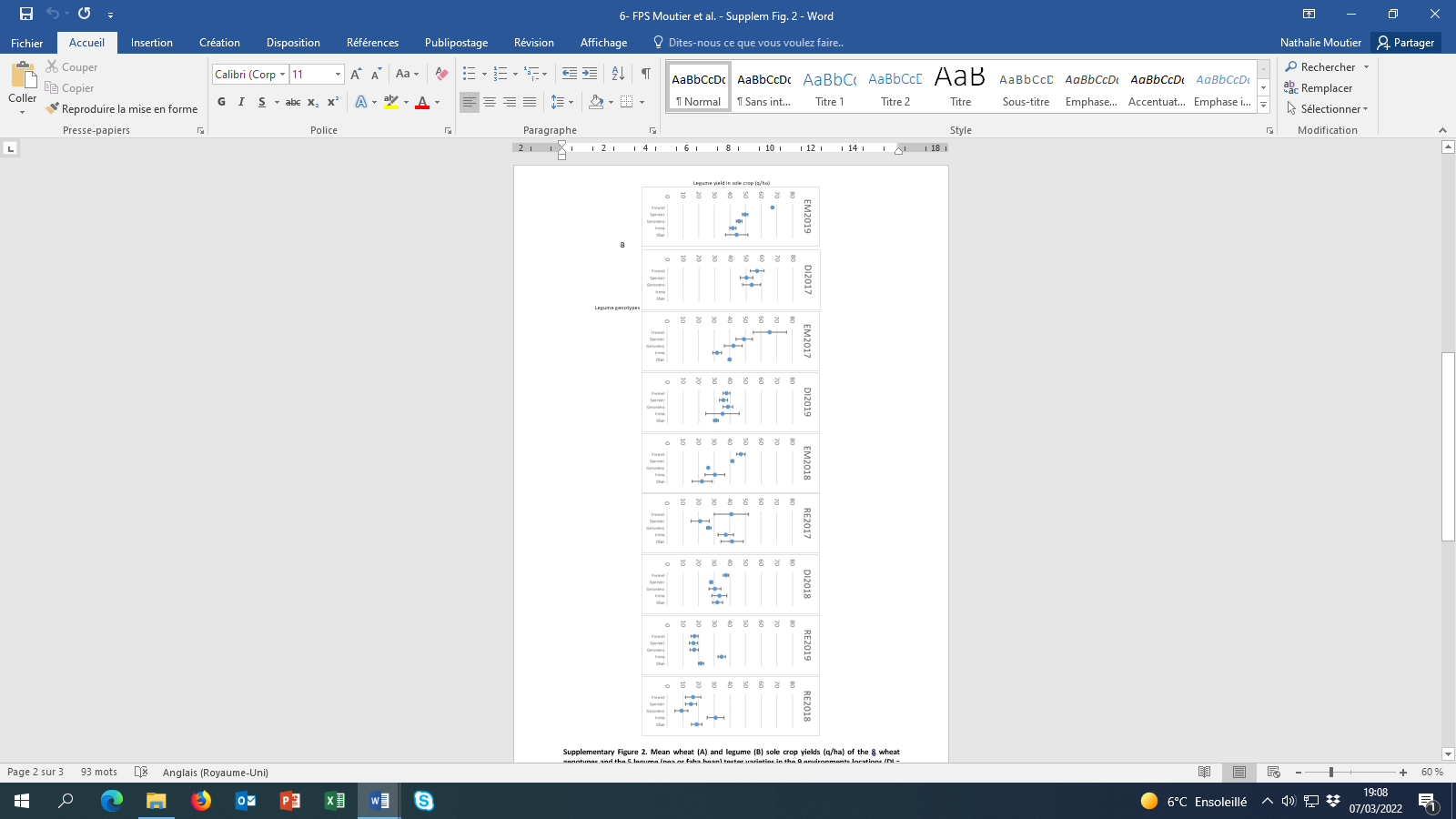 |
| --- | --- |

**Supplementary Figure 2. Mean wheat (A) and legume (B) sole crop yields (q/ha) of the 8 wheat genotypes and the 5 legume (pea or faba bean) tester varieties in the 9 environments locations (DI = INRAE Dijon, EM = INRAE Estrées-Mons, RE = INRAE Rennes; years 17=2017, 18=2018, 19=2019). Environments are classified from the highest to the lowest yield for all genotypes. Genotypes are classified from the highest to the lowest yield across all environments.**
